# Molecular typing of mycobacterium tuberculosis isolates circulating in Jiangsu Province, China

**DOI:** 10.1186/1471-2334-11-288

**Published:** 2011-10-26

**Authors:** Qiao Liu, Dandan Yang, Weiguo Xu, Jianming Wang, Bing LV, Yan Shao, Honghuan Song, Guoli Li, Haiyan Dong, Kanglin Wan, Hua Wang

**Affiliations:** 1 Department of Epidemiology and Biostatistics, School of Public Health, Nanjing Medical University, Nanjing, PR China; 2 Department of Chronic Communicable Disease, Center for Disease Control and Prevention of Jiangsu Province, Nanjing, PR China; 3 National Institute for Communicable Disease Control and Prevention, Chinese Center for Disease Control and Prevention, State Key Laboratory for Infectious Disease Prevention and Control, Beijing, PR China

## Abstract

**Background:**

Globally, China is the second place with high burden of tuberculosis (TB). To explore the characteristics of the pathogens of *Mycobacterium tuberculosis *(MTB) circulating in this area is helpful for understanding and controlling the spread of the strains. Recent developments in molecular biology have allowed prompt identification and tracking specific strains of MTB spreading through the population.

**Methods:**

Spacer-oligonucleotide typing (spoligotyping) and mycobacterial interspersed repetitive units variable number tandem repeat (MIRU-VNTR) were performed in combination to yield specific genetic profiles of 260 MTB strains isolated from 30 counties of Jiangsu province in China between June and July 2010. The spoligotyping results were in comparison to the world Spoligotyping Database of Institute Pasteur de Guadeloupe (SpolDB4). Drug susceptibility test (DST) was performed on all strains by proportion method on Lowenstein-Jensen (LJ) culture media.

**Results:**

Based on the spoligotyping method, 246 strains displayed known patterns and 14 were absent in the database. Predominant spoligotypes belonged to the Beijing family (80.4%). By using the 24-loci VNTR typing scheme, 224 different patterns were identified, including 20 clusters and 204 unique patterns. The largest clade comprised 195 strains belonging to the Beijing family. The combination of spoligotyping and 24-loci MIRU-VNTR demonstrated maximal discriminatory power. Furthermore, we observed a significant association between Beijing family strains and drug-resistant phenotypes. The Beijing family strains presented increased risks for developing multi-drug resistant TB, with the OR (95% CI) of 11.07(1.45-84.50).

**Conclusions:**

The present study demonstrated that Beijing family isolates were the most prevalent strains circulating in Jiangsu province of China. The utility of spoligotyping in combination with 24-loci MIRU-VNTR might be a useful tool for epidemiological analysis of MTB transmission.

## Background

Tuberculosis (TB) has been a global threat to human health and it remains a major public health burden in developing countries. As a public health dilemma, drug resistance has been an obstacle to achieve the goal of effective TB control. In 2009, it was reported that about 9.4 million incident cases (8.9 million-9.9 million) occurred globally and among them, 250 000 (230 000-270 000) were multi-drug resistant TB (MDR-TB) [1]. China is one of the five countries with the largest number of both incident cases (1.1-1.5 million) and MDR-TB cases (79 000-120 000) in absolute terms in 2008 [1]. The increased rate of drug-resistance and MDR strains of TB remain a serious problem of TB control in China.

MDR-TB may arise by inadequate antituberculosis treatment or by direct transmission of drug resistant strains from one individual to another [2]. The association with MDR-TB has led to concerns that *Mycobacterium tuberculosis *(MTB) strains may have a predilection for acquiring drug resistance. DNA fingerprinting of MTB isolates is a powerful tool to study the molecular epidemiological characteristics of MDR-TB. The traditional method used to genotype MTB complex (MTBC) is insertion sequence (IS) 6110 restriction fragment-length polymorphism (RFLP), which can be utilized in outbreak investigations, long-term surveillance and the detection of laboratory contamination. Despite the widespread use of IS6110-based RFLP analysis, it remains a labour intensive, technically demanding, and insufficiently discriminating strains with the low copy numbers (fewer than six) of IS6110 [3]. Following IS6110-based RFLP, spoligotyping has been the most widely used method for secondary typing of MTB strains and it has been particularly useful for identifying strains belonging to the Beijing/W family due to the absence of spacers 1-34 in the direct repeat (DR) region of the MTB genome [4]. Variable number tandem repeat (VNTR) is a newly developed typing method, which determines the number of repeated mycobacterial interspersed repetitive units (MIRU) by using small quantities of bacteria [5]. MIRU-VNTR has shown a higher discriminatory power when combining with spoligotyping. The advantages of such PCR-based genotyping techniques include their easy digitalization of the generated profiles and hence easy inter-laboratory comparison, as well as easy creation and maintenance of the database [6].

The Beijing family, first identified in 1995 in Beijing of China, almost omnipresent and significantly prevalent in certain world regions, e.g., East Asia and the former Union of Soviet Socialist Republics(USSR) [7]. Several studies have observed that the Beijing family MTB strains had exhibited important pathogenic features which might be associated with the drug resistant TB in China [6,8-10]. As the prevalence of drug resistant clones of MTB varies from one area to another, studies in geographical distribution of resistant clones are helpful for understanding the epidemiological characteristics of TB.

Jiangsu is a province locating along the eastern coast of China and it covers an area of 102.6 thousand square kilometers, with a total population of 77 million in 2009. The high prevalence of drug resistance among TB patients in Jiangsu province has been a major challenge for TB control. According to the most recently updated data, the proportion of primary and acquired MDR-TB were 7.63% and 33.7%, respectively [11].

The main goal of this study was to characterize the genotyping of MTB strains circulating in Jiangsu Province and to explore whether there is any evidence that the spreading of Beijing family of MTB strains was associated with the drug resistance.

## Methods

### Mycobacterial strains

The study was conducted in 30 counties of Jiangsu province, which were sampled according to the "Guidelines for surveillance of drug resistance in tuberculosis" developed by WHO/IUATLD [12]. Two hundred and sixty newly diagnosed sputum smear positive pulmonary TB patients from June 2010 to July 2010 in the study sites were recruited. Totally, there were 216 new cases and 44 previously treated ones. The average age of cases was 48.5 years old. All TB patients were diagnosed by referring to the national guidelines of China and reviewed by Jiangsu provincial Center for Disease Control and Prevention. Each subject present three sputum smear samples with labeled plastic bottles for sputum smear microscopy test. The two sputum samples with the highest bacterial counts were cultured, and one culture was submitted to the provincial reference laboratory for drug susceptibility test (DST). Sputum smear microscopy and culture were performed at the level of county (district) laboratory.

### Strains isolation and drug susceptibility test

The sputum samples were cultured and isolated on Lowenstein-Jensen (LJ) culture media. LJ medium was impregnated with isoniazid (INH), rifampicin (RIF), streptomycin (SM), and ethambutol (EMB). The concentrations of anti-tuberculosis drugs were 0.2 μg/ml for INH, 40 μg/ml for RIF, 4 μg/ml for SM, and 2 μg/ml for EMB. The strain was declared resistant to the specific drug; or it was defined as sensitive when the growth rate was < 1% compared to the control. MDR-TB was defined as strains being resistant to at least RIF and INH [11]. Strains isolation, identification and drug susceptibility test (DST) were performed at the provincial reference laboratory.

### Genomic DNA extraction

Mycobacterial genomic DNA was extracted from mycobacterial colonies growing on LJ medium by resuspending one loop of mycobacterial colonies in 200 μl TE buffer (10 mM Tris-HCl, 1 mM EDTA) and was incubated at 85°C for 30 minutes to obtain genomic DNA. After centrifugation of the suspension, the supernatant fluid containing DNA was removed and stored at -20°C until further use [13]. Laboratory strain MTB H37Rv was used as a control for all microbiological and genetic procedures.

### Genotyping

Spoligotyping of the isolates was performed as described by Kamerbeek et al [4]. In brief, genomic DNA of MTB isolates were amplified using polymerase chain reaction (PCR) with the primers of Dra (biotin labelled) and Drb, and then the PCR products were hybridized to a set of 43 oligonucleotide probes corresponding to each spacer, which were covalently bound to a membrane [4]. Spoligotypes in binary format were compared with the SpolDB4 database. In particular, strains with spoligotype patterns characterized by deletion of spacers 1-34 were defined as "typical" Beijing genotypes, whereas strains with additional deletion of one or more of the last nine spacers were defined as Beijing-like genotypes according to the criteria of the international database SpolDB4 [4,14-16].

To identify a suitable MIRU-VNTR loci set for genotyping MTB in this area, the number of tandem repeats was determined in 24 MIRU-VNTR genetic loci: ten original MIRU-VNTR loci; six loci of exact tandem repeats (ETRs): ETR-A, -B, -C, -D, -E and -F; five Mtub loci: Mtub4, 21, 30, 38, and 39; and three Queen's University of Belfast (QUBs) loci: QUB-11b, -26, and 4156c. PCR amplification was performed for correspondent loci as described in the previous studies [17-20]. PCR products were analyzed on a 2% agarose gel against a 100-bp DNA ladder, and the copy number at each locus was calculated using the Quantity 1 gel imaging system [10]. Determination of the discriminatory power of the VNTR loci was calculated using the Hunter-Gaston discriminatory Index (HGDI) [21]. The HGDI was calculated using the following formula:

$HGDI=1-\left[\frac{1}{N\left(N-1\right)}\overset{s}{\underset{j=1}{\sum }}nj\left(nj-1\right)\right]$, where D is the numerical index of discrimination, N is the total number of strains in the typing scheme, *s *is the total number of different strain types, and *nj *is the number of strains belonging to the *j*th type.

### Data analysis

Spoligotypes in binary format were entered in an Excel spreadsheet and compared with the spoligotyping database SpolDB4. The patterns were established clusters in the BioNumerics software version 5.0 (Applied Maths, Sint-Martens-Latem, Belgium). The strength of association between genotypes of MTB strains and drug resistance was estimated by odds ratio (OR) and 95% confidence interval (95% CI). All tests of significance were two sided and a significant threshold was set at 0.05. Statistical analyses were carried out by using SPSS software 13.0 (SPSS Inc., USA).

### Ethical consideration

This project has been approved by Institutional Review Board of Nanjing Medical University. Written informed consent was obtained from all participants. Ethics has been respected throughout the whole study period.

## Results

### Spoligotyping analysis

**Figure 1:**
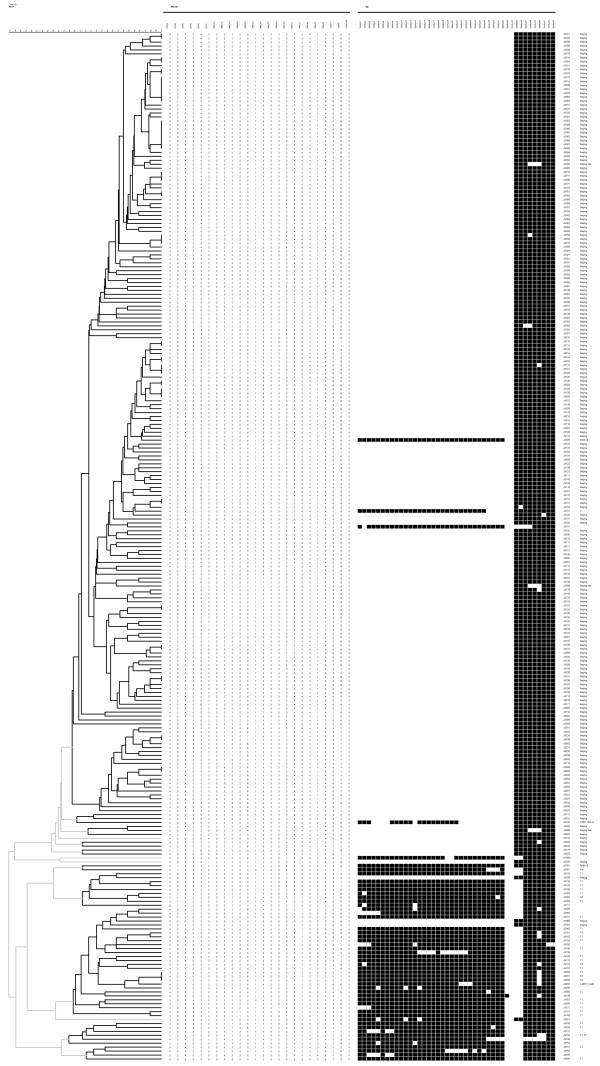
**Samples with MIRU-VNTR and spoligotyping**. Clustering was based upon an average of MIRU-VNTR and spoligotyping, clustered using the dice coefficient and the unweighted pair group method with arithmetic averages (UPGMA) in BioNumerics 5.0.

Reproducible results were obtained for 260 strains and 34 distinct spoligotype clusters were obtained as referring to the SpolDB4.0 database. Twenty-seven spoligotypes represented the single isolate and the other 233 isolates were grouped into 7 clusters containing from 2 to 199 isolates (Figure 1). Among them, 14 (5.4%) strains were found to be undefined (absent in the SpolDB4.0 database) while 246(94.6%) were successfully clustered by spoligotyping and divided into 11 types (STs). Out of these, 5 STs had 2 or more strains whereas 6 STs had single isolate each. A determination of predominant STs were Beijing family (n = 209, 80.4%), T1 (n = 23, 8.8%) and T2 (n = 6, 2.3%), representing about 91.5% of total strains (Table 1).

**Table 1:** Spoligotypes shared by *Mycobacterium tuberculosis *strains evaluated in this study

**S.NO**.	Family	N(%)	Binary
1	Beijing	206(79.2)	□□□□□□□□□□□□□□□□□□□□□□□□□□□□□□□□□□■■■■■■■■■
2	Beijing-like	3(1.2)	□□□□□□□□□□□□□□□□□□□□□□□□□□□□□□□□□□■■■□□□■■■
3	T1	23(8.8)	■■■■■■■■■■■■■■■■■■■■■■■■■■■■■■■■□□□□■■■■■■■
4	T2	6 (2.3)	■■■■■■■■■■■■■■■■■■■■■■■■■■■■■■■■□□□□■■■□■■■
5	MANU2	2(0.8)	■■■■■■■■■■■■■■■■■■■■■■■■■■■■■■■■□□■■■■■■■■■
6	U	1(0.4)	■■■■■■■■■■■■■■■■■■■■■■■■■■■■■■■■■■■■■■■■■■■
7	T1-T2	1(0.4)	■■■■■■■■■■■■■■■■■■■■■■■■■■■■■■■■□□□□■■■□□■■
8	H3	1(0.4)	■■■■■■■■■■■■■■■■■■■■■■■■■■■■■■□■□□□□■■■■■■■
9	H4	1(0.4)	■■■■■■■■■■■■■■■■■■■■■■■■■■■■□□□■□□□□■■■■■■■
10	LAM10_CAM	1(0.4)	■■■■■■■■■■■■■■■■■■■■■■□□□■■■■■■■□□□□■■■□■■■
11	CAS1_DELHI	1(0.4)	■■■□□□□■■■■■□■■■■■■■■■□□□□□□□□□□□□■■■■■■■■■
12	Undefined	1(0.4)	■□■■■■■■■■■■□■■■■■■■■■■■■■■■■■■■□□□□■■■■■■■
13	Undefined	1(0.4)	■■■■■■■■■■■■■■■■■■■■■■■■■■■■■■■■■□□□■■■□■■■
14	Undefined	1(0.4)	■■■■□■■■■■■■□■■■■■■■■■■■■■■■■■■■□□□□■■■■■■■
15	Undefined	1(0.4)	□■■■■■■■■■■■□■■■■■■■■■■■■■■■■■■■□□□□■■■□■■■
16	Undefined	1(0.4)	■□■■■■■■■■■■■■■■■■■■■■■■■■■■■■■■□□□□□□■■■■■
17	Undefined	1(0.4)	■■■■□■■■■■□■■□■■■■■■■■■■■■■■■■■■□□■■■■■■■■■
18	Undefined	1(0.4)	■■■■□■■■■■□■■□■■■■■■■■■■■■■■■■■■□□□□■■■■■■■
19	Undefined	1(0.4)	□□□□□■■■■■■■■■■■■■■■■■■■■■■■■■■■□□□□■■■■■■■
20	Undefined	1(0.4)	□□□■■■■■■■■■□■■■■■■■■■■■■■■■■■■■□□□□■■■■■□□
21	Undefined	2(0.8)	■■□□□■□□■■■■■■■■■■■■■■■■■■■■■■■■□□□□■■■■■■■
22	Undefined	1(0.4)	■■■■■■■■■■■■■■■■■■■■■■■■■■■■□□□□□□□□□□□□□■■
23	Undefined	1(0.4)	■■■■■■■■■■■■■■■■■■■□□□□□■□■□■■■■□□□□■■■■■■■
24	Undefined	1(0.4)	■■■■■■■■■■■■■□□□□■□□□□□□■■■■■■■■□□□□■■■■■■■

(■) Presence of spacer; (□) absence of spacer. N = number of strains.

### MIRU-VNTR

Among 260 MTB strains being genotyped, 224 different VNTR genotypes were detected. Two hundred and four (91.1%) were unique (i.e., observed for only one strain) and 56 strains could be grouped into 20 clusters, each including 2 to 8 strains (Additional file 1). Two main clusters which contained 8 (14.3%) and 7 (12.5%) strains showed 4 2 4 3 4 3 2 3 3 2 5 1 5 3 3 3 4 5 4 1 4 5 6 3 and 4 2 4 3 4 3 2 3 3 2 5 1 5 3 3 3 4 5 4 1 4 6 6 3 VNTR profiles, respectively (Figure 1). HGDI scores which were calculated for particular MIRU loci varied significantly from 0.717 of Qub11b to 0 of MIRU24. As suggested by Sola et al [22], the MIRU loci were further classified into highly (> 0.6), moderately (0.3 to 0.6), and poorly (< 0.3) discriminating based on the HGDI scores. Five loci (ETRB, ETRC, MIRU20, MIRU02, MIRU24) showed negligible diversity (HGDI < 0.1) whereas ETRE and Qub11b had highly discriminating ability, especially for Beijing family (Table 2).

**Table 2:** Variability of 24-VNTR loci among Mycobacterium tuberculosis strains (n = 260)

Alias	HGDI (All strains)	HGDI (Beijing family)	12-loci VNTR	NO. of alleles observed	Size range observed
Qub11b	0.717	0.629	√	8	1-8
ETRE	0.695	0.668	√	5	2-6
MIRU26	0.683	0.560	√	7	1-7
Mtub21	0.660	0.535	√	8	1-9
Qub26	0.646	0.613	√	9	1-9
ETRD	0.592	0.536	√	6	1-6
Mtub04	0.467	0.426	√	4	2-5
MIRU40	0.454	0.276	√	4	1-4
MIRU10	0.446	0.262	√	5	1-5
MIRU39	0.390	0.178	√	4	1-4
Mtub39	0.386	0.213	√	7	2-8
Mtub30	0.365	0.196	√	4	2-5
MIRU16	0.348	0.262		4	1-4
ETRF	0.343	0.243		4	1-4
Mtub38	0.335	0.065		4	1-4
ETRA	0.313	0.201		4	2-5
MIRU23	0.268	0.250		5	3-7
MIRU27	0.243	0.084		4	1-4
Qub4156c	0.179	0.203		5	1-5
ETRB	0.096	0.056		3	1-3
ETRC	0.090	0.066		5	2-6
MIRU20	0.023	0.010		2	1-2
MIRU02	0.008	0.010		2	1-2
MIRU24	0	0		1	1

### Drug-susceptibility patterns of the MTB isolates

A total of 260 MTB strains isolated from the sputum samples of TB patients were used for drug susceptibility test. Among them, 13.1% (34/260) were MDR-TB strains, which were resistant to at least INH and RIF, the two most powerful antituberculosis drugs. Furthermore, we compared the distribution of drug resistance between Beijing and non-Beijing genotyping strains. As shown in table 3, among non-Beijing family strains, the proportion of mono-drug resistance was 7.8% (n = 4) to INH, 2.0% (n = 1) to RIF, 9.8% (n = 5) to SM, while 19.6% (n = 10) were resistant to at least one drug and 2.0% (n = 1) were MDR-TB. For Beijing family strains, the proportion of mono-drug resistance was 3.3% (n = 7) to INH, 1.4% (n = 3) to RIF, 5.7% (n = 12) to SM and 0.5% (n = 1) to EMB, respectively, while 11.0% (n = 23) were resistant to at least one drug and 15.8% (n = 33) were MDR-TB. A binary logistic regression model was applied to analyze the factors associated with MDR-TB. The Beijing family strains presented increased risks for developing MDR (15.8% vs.2.0%, OR: 11.07, 95%CI: 1.45-84.50) (Table 4).

**Table 3:** Differences of characteristics between Beijing and non-Beijing family

Factors	Cases n = 260	Non-Beijing family n = 51, n(%)	Beijing family n = 209, n(%)	χ^2 ^	*P*
**Sex**					
Men	180(69.2)	37(72.5)	143(68.4)		
Women	80(30.8)	14(27.5)	66(31.6)	0.328	0.567
**Treatment history**					
No	216(83.1)	41(80.4)	175(83.7)		
Yes	44(16.9)	10(19.6)	34(16.3)	0.325	0.568
**Pan-susceptible cases**	186(71.5)	39(76.5)	147(70.3)	0.758	0.384
**Any drug-resistant cases**	74(28.5)	12(23.5)	62(29.7)	0.758	0.384
**Mono-resistance**					
INH	11(4.2)	4(7.8)	7(3.3)		0.234*
RIF	4(1.5)	1(2.0)	3(1.4)		0.585*
SM	17(6.5)	5(9.8)	12(5.7)		0.340*
EMB	1(0.4)	0	1(0.5)		1*
Total	33(12.7)	10(19.6)	23(11.0)	2.738	0.098
**MDR-TB^#^**					
INH+RIF	6(2.3)	0	6(2.9)		0.601*
INH+RIF+SM	9(3.5)	0	9(4.3)		0.212*
INH+RIF+EMB	2(0.8)	0	2(1.0)		1*
INH+RIF+SM+EMB	17(6.5)	1(2.0)	16(7.7)		0.208*
MDR	34(13.1)	1(2.0)	33(15.8)	6.897	**0.009**

INH, Isoniazid; RIF, Rifampicin; EMB, Ethambutol; SM, Streptomycin.

#Defined as resistant to at least INH and RIF.

*Value by Fisher's exact test.

**Table 4:** Factors associated with multi-drug resistant tuberculosis

Factors	Cases	No-MDR	MDR	cOR(95% CI)	*P*	aOR(95% CI)	*P*
	n = 260	n = 226, n(%)	n = 34, n(%)				
**Gender**							
Men	180	155(68.6)	25(73.5)	1		1	
Women	80	71(31.4)	9(26.5)	0.79(0.35-1.77)	0.561	0.81(0.34-1.96)	0.643
**Age(years)**							
< 30	66	56(24.8)	10(29.4)	1		1	
30-	111	93(41.2)	18(52.9)	1.08(0.47-2.51)	0.851	0.68(0.23-1.99)	0.479
60-	57	51(22.6)	6(17.6)	0.66(0.22-1.94)	0.449	0.36(0.08-1.71)	0.199
75+	26	26(11.5)	0	0	0.998	0	0.998
**Treatment history^#^**							
No	216	194(85.8)	22(14.2)	1		1	
Yes	44	22(64.7)	12(35.3)	3.31(1.49-7.34)	0.002	3.39(1.37-8.41)	0.008
**BCG vaccination**							
No	142	125(55.3)	17(50.0)	1		1	
Yes	118	101(44.7)	17(50.0)	1.24(0.60-2.55)	0.562	0.81(0.26-2.48)	0.711
**Migrant population**							
No	174	151(66.8)	23(67.6)	1		1	
Yes	86	75(33.2)	11(32.4)	0.96(0.45-2.08)	0.923	0.94(0.40-2.23)	0.894
**Beijing family**							
No	51	50(22.1)	1(2.9)	1		1	
Yes	209	176(77.9)	33(97.1)	9.38(1.25-70.26)	**0.009**	11.07(1.45-84.50)	**0.023**

cOR, crude odds ratio; aOR, adjusted odds ratio, adjusted for sex, age, treatment history, BCG vaccination, migrant population, and Beijing family; #:Those having previously received anti-tuberculosis treatment for more than one month was defined as previously treated cases.

## Discussion

This study demonstrates that the Beijing genotype is the most predominant lineage of TB strains in Jiangsu province, which is consistent to the findings from other areas of China. As described in table 5, Beijing family accounts for 85.12% to 92.59% of the MTB strains currently epidemic in the Beijing area [23,24] and it is also prevalent in Heilongjiang (89.50%) [10], Gansu (87.50%) [24], Jilin (89.88%) [24], Ningxia (67.12%) [25], Henan (80.00%) [24], Shandong (85.93%) [9], Shanxi (80.00%) [24], Tianjin(91.07%) [26], Tibet (90.38%) [24], Xinjiang (67.80%) [24], Anhui (85.35%) [24], Fujian (54.50%) [24], Hong Kong (68.45% and 70.00%) [27,28], Hunan (66.00%) [24], Jiangsu and Zhejiang (69.23%) [29], Shanghai (77.14%) [30], Zhejiang (64.84%) [24], Guangxi (55.29%) [24], Taipei (52.53%) [13], and Sichuan (57.89% to 63.64%) [24,31]. It was found that amongst all MTB strains studied, the "Beijing" genotype strains were highly prevalent in our geographic area. The proportion of Beijing family circulating in the north area (83.91%) is higher than that in the south area (66.40%) of China (*P *< 0.001).

**Table 5:** Distribution of Beijing family strains in China

Area	Strains tested	Beijing family strains	Proportion (%)	Location
Beijing [23]	121	103	85.12	north
Beijing[23,24]	108	100	92.59	north
Gansu [24]	224	196	87.50	north
Heilongjiang [10]	200	179	89.50	north
Henan [24]	95	76	80.00	north
Jilin [24]	326	293	89.88	north
Ningxia [25]	72	49	67.12	north
Shandong [9]	135	116	85.93	north
Shanxi [24]	115	92	80.00	north
Tianjin [26]	112	102	91.07	north
Tibet [24]	208	188	90.38	north
Xinjiang [24]	205	139	67.80	north
Anhui [24]	157	134	85.35	south
Fujian [24]	433	236	54.50	south
Hong Kong [27]	355	243	68.45	south
Hong Kong [28]	500	350	70.00	south
Hunan [24]	100	66	66.00	south
Jiangsu and Zhejiang [29]	351	243	69.23	south
Zhejiang [24]	91	59	64.84	south
Shanghai [30]	175	135	77.14	south
Guangxi [24]	208	115	55.29	south
Taipei [13]	356	187	52.53	south
Sichuan [24]	76	44	57.89	south
Sichuan [31]	143	91	63.64	south
Jiangsu	260	209	80.38	south

Though world widely the Beijing genotype is apparently the second most prevalent genotype, following after AFR lineage [16], reasons for its successful global spread remain poorly understood. The infective success of this lineage seems to be associated with its effect on the immune response, in that it can control the release of the macrophage-derived cytokines that play a central role in directing the immune response towards a non-protective Th2 phenotype [32,33]. The widely distributed (but not universal) association of drug resistance and the Beijing genotype suggests that these strains may have a particular propensity for acquiring drug resistance [14]. Besides China, they are increasingly reported in other areas of the world and are frequently associated with outbreaks of TB or MDR-TB [14,29,34-37], but not all [9,10,27,38,39]. In the present study, we observed a significant difference of drug resistance between Beijing and non-Beijing strains, where the Beijing family was associated with an increased risk of MDR-TB. These data are accordant with the results from studies so far performed in China [29,40]. Definite conclusions on the extent of spread and associations with drug resistance, however, could not yet be drawn. This was due to the limited amount of information available from most areas of the world, the possible biases in many of the published reports, and the absence of standard definitions and study designs.

Both spoligotyping and MIRU-VNTR have different abilities to discriminate MTB strains and can be performed in combination. For example, among 260 strains included in this analysis, 209 (80.4%) were classified as the Beijing genotype. The majority of these Beijing genotype isolates showed the typical spoligotype pattern (hybridization to all of spacers 35-43 and no hybridization to spacers 1-34). Four strains showed the non-Beijing genotype spoligotype pattern, but had specific Beijing genotype signatures of MIRU-VNTR. Furthermore, another four strains showing the typical Beijing genotype spoligotype pattern, but can't be distinguished by MIRU-VNTR (Figure 1). One possible explanation might be the multiple infections of strains in these eight samples [41-43], however, it is rarely found when using the DNA fingerprinting method. Till now, no definition of the Beijing genotype on the basis of genetic markers will be 100% perfect, because there will always be exceptional strains [14]. As shown in our study, when 209 strains in the Beijing clade identified by spoligotyping were further discriminated by MIRU-VNTR, nearly all (205/209, 98.1%) Beijing strains were parts of a clade (Figure 1). This study further demonstrated that MTB strains grouped into the Beijing family by spoligotyping had similar patterns of MIRU-VNTR. This was borne out by the fact that all MIRU-VNTR patterns of Beijing family strains were highly similar.

Spoligotyping has been proved to be the most useful method to recognize Beijing lineage strains as it is the most rapid and easiest method to apply and it correlates well with the other methods [14]. But this method had a poor discriminability when it was used independently, thus should be performed in combination with another independent method (for example MIRU-VNTR typing) to allow effective epidemiological investigation [22]. As shown in the present study, spoligotyping alone yielded 34 genotypes, 7 clusters, with 27 unique genotypes and a HGDI of 0.409. MIRU-VNTR clustered 21.5% of strains with an HGDI of 0.998. The combination of spoligotyping and MIRU-VNTR typing in our population clustered 19 of all 260 strains. Different VNTR typing sets showed various efficiencies in different Beijing genotype strains (Table 6). The discriminatory power of the traditional 12 loci MIRU-VNTR has been found to be insufficient [5], which is often failed to differentiate clonal strains in areas where the Beijing family genotype is predominant [27]. Other sets of MIRU-VNTR loci, such as an optimized set of 24 loci, have also been defined. However, all 24 loci are not required for MTB genotyping in any given situation [5], as the number of loci required depends on the lineage known to be prevalent in the investigated area. We have evaluated MIRUs loci, QUB loci and ETR loci individually for their abilities to differentiate the Beijing and non-Beijing genotype families of MTB. When the top 12 loci (MIRU26,40,10, 39; QUB11b,26; Mtub21,04,39,30; ETR E,D) were selected for typing, the set produced a HGDI value of 0.996 in all stains and 0.994 in Beijing family stains, which was almost equal to that obtained in 24 loci MIRU-VNTR (0.998 and 0.997). This optimized 12 loci VNTR typing set could be an important tool for tracking MTB of the Beijing family in this area.

**Table 6:** Comparative analysis of genotyping methods applied to *Mycobacterium tuberculosis *strains (n = 260)

**Typing method**, strain groups	Genotypes in total	Unique genotypes	clusters	Strains in cluster	HGDI value
**Spoligotyping**					
All strains	34	27	7	2-199	0.409
Beijing family only	7	4	3	3-196	0.093
Non-Beijing strains	27	23	4	2-19	0.856
**MIRU-VNTR analysis, All strains**					
24-VNTR analysis	224	204	20	2-8	0.998
12-VNTR analysis	204	177	27	2-9	0.996
**Spoligo+24-VNTR**					
All strains	227	208	19	2-8	0.998
Beijing family only	178	160	18	2-8	0.997

As observed by 24-MIRU-VNTR analysis, it appeared that individuals infected with strains belonging to the Beijing genotype were more likely to be part of a cluster. Beijing strains were isolated from patients from different counties of Jiangsu Province, suggesting that these virulent strains are spreading throughout the province. In addition to Beijing family strains, we also detected strains belonging to other families such as T1, T2, H3, H4, CAS, LAM, U, and MANU2. The CAS family was previously found primarily in India [44], and also detected in Tibet and Xinjiang [24]. The LAM family was predominantly prevalent in South America and West-Africa, and also found in Taiwan of China [13]. It can be suggested that strains of these families have started spreading across Jiangsu province; of course, this requires further testing using more extended typing of more clinical strains.

Future studies are needed to clarify the characteristics of Beijing family, one important genotype of MTB. Possible variations in host-pathogen interactions among strains of various genotypes needs to be identified and the role of Beijing genotype infection as a possible risk factor for drug resistance and/or treatment failure must urgently be addressed in longitudinal studies in the affected high incident regions. In short, the effect of this genotype on TB control efforts need to be further investigated.

## Conclusion

This is the first report on the genotypes of MTB stains using spoligotyping in combination with 24-loci MIRU-VNTR technology in Jiangsu province of China. Based on our preliminary data, Beijing family strains have predominantly prevalent in Jiangsu and shown a high number of clusters in the study population. Furthermore, we also observed that the high prevalence of Beijing genotype may be associated with the risk of MDR-TB. Further genome level studies will be needed to investigate the molecular specificities of the major subgroup.

## Competing interests

The authors declare that they have no competing interests.

## Authors' contributions

QL, HW, WX conceived the study, carried out the molecular genetic studies, analyzed the data and drafted the manuscript; DY, YS, HS, GL participated in the study design, implemented the field investigation and performed DST tests; BL and YD participated in the genotyping analysis. KW and JW participated in the study design and helped draft the manuscript. All authors contributed to the study and have read and approved the final manuscript.

## Pre-publication history

The pre-publication history for this paper can be accessed here:

http://www.biomedcentral.com/1471-2334/11/288/prepub

## Supplementary Material

Additional file 1 **MIRU-VNTR genotypes of *Mycobacterium tuberculosis *isolates**. Clustering was based upon an average of MIRU-VNTR, clustered using the categorical co-efficient and UPGMA in BioNumerics 5.0. Click here for file
