# Supplementary figures and images for: Molecular typing of mycobacterium tuberculosis isolates circulating in Jiangsu Province, China
Source: BMC Infect Dis. 2011 Oct 26;11:288. doi: 10.1186/1471-2334-11-288 (PMC3215657; doi:10.1186/1471-2334-11-288)

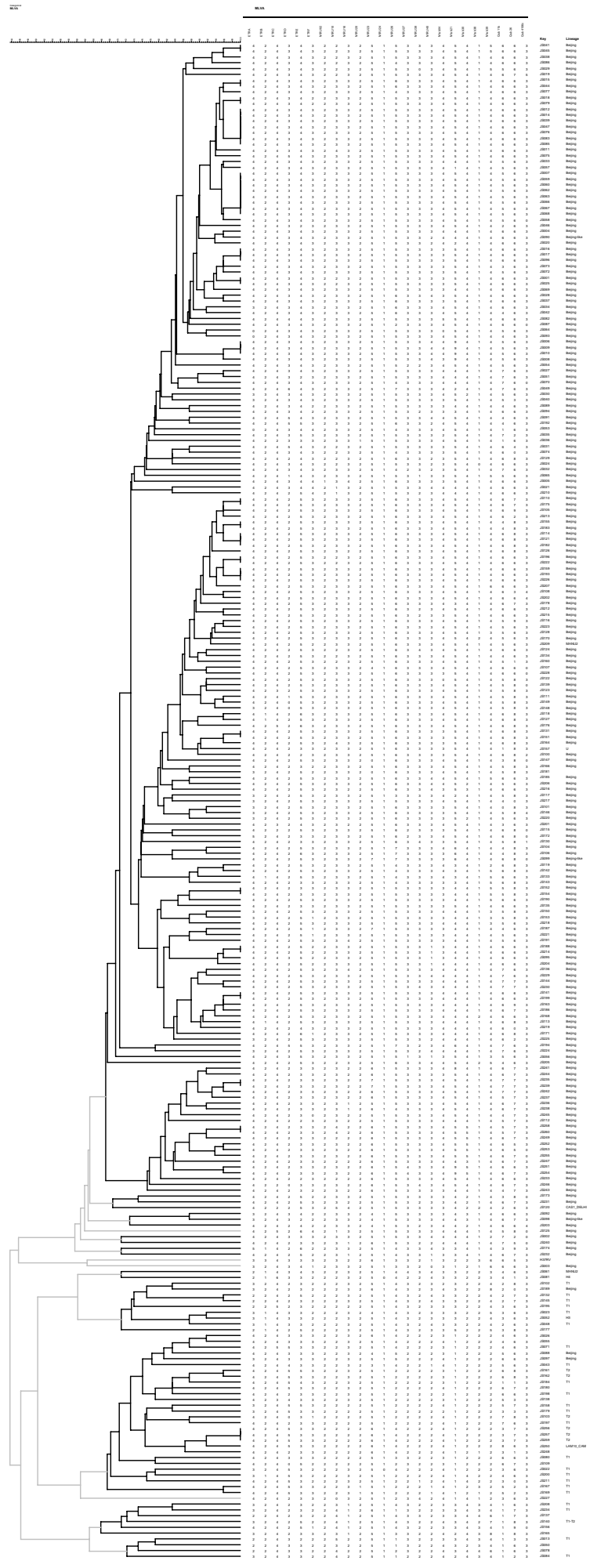

Supplement: Additional file 1 — MIRU-VNTR genotypes of Mycobacterium tuberculosis isolates. Clustering was based upon an average of MIRU-VNTR, clustered using the categorical co-efficient and UPGMA in BioNumerics 5.0. [file 1471-2334-11-288-S1.PDF]
